# Monitoring of inflammation using novel biosensor mouse model reveals tissue- and sex-specific responses to Western diet

**DOI:** 10.1242/dmm.049313

**Published:** 2022-06-24

**Authors:** Sarah Talley, Raiza Bonomo, Chaitanya Gavini, Jomana Hatahet, Emily Gornick, Tyler Cook, Byeong Jae Chun, Pete Kekenes-Huskey, Gregory Aubert, Edward Campbell, Virginie Mansuy-Aubert

**Affiliations:** 1 Department of Microbiology and Immunology, Stritch School of Medicine, Loyola University Chicago, 2160 S. First Avenue, Maywood, IL 60153, USA; 2 Department of Cell and Molecular Physiology, Stritch School of Medicine, Loyola University Chicago, 2160 S. First Avenue, Maywood, IL 60153, USA; 3 Department of Internal Medicine, Division of Cardiology, Stritch School of Medicine, Loyola University Chicago, 2160 S. First Avenue, Maywood, IL 60153, USA

**Keywords:** Caspase, Gut microbiome, Inflammasome, Mouse biosensor, Obesity, Western diet

## Abstract

Obesity is an epidemic, and it is characterized by a state of low-grade systemic inflammation. A key component of inflammation is the activation of inflammasomes, multiprotein complexes that form in response to danger signals and that lead to activation of caspase-1. Previous studies have found that a Westernized diet induces activation of inflammasomes and production of inflammatory cytokines. Gut microbiota metabolites, including the short-chain fatty acid butyrate, have received increased attention as underlying some obesogenic features, but the mechanisms of action by which butyrate influences inflammation in obesity remain unclear. We engineered a caspase-1 reporter mouse model to measure spatiotemporal dynamics of inflammation in obese mice. Concurrent with increased capsase-1 activation *in vivo*, we detected stronger biosensor signal in white adipose and heart tissues of obese mice *ex vivo* and observed that a short-term butyrate treatment affected some, but not all, of the inflammatory responses induced by Western diet. Through characterization of inflammatory responses and computational analyses, we identified tissue- and sex-specific caspase-1 activation patterns and inflammatory phenotypes in obese mice, offering new mechanistic insights underlying the dynamics of inflammation.

## Abstract (precis)

**Summary:** We used a caspase-1 reporter mouse model to measure spatiotemporal dynamics of inflammation in obese mice, identifying tissue- and sex-specific caspase-1 activation patterns and inflammatory phenotypes, indicating mechanisms underlying the dynamics of inflammation.

## INTRODUCTION

Obesity (body-mass index ≥30 kg/m^2^) is the result of long-term energy homeostasis imbalance (e.g. increased food intake, decreased energy expenditure) involving brain and peripheral organs (Spiegelman and Flier, 2001). Obesity is the cause of many complications, including diabetes, liver diseases, cardiovascular diseases, or even neurological dysfunction such as memory impairment or pain (Grundy et al., 2004; O'Brien et al., 2017). Low-grade inflammation is thought to play a major role in the onset and progression of obesity and its complications (Hotamisligil, 2006, 2017a,b). Inflammasomes are multiprotein complexes that have important functions in innate immunity and serve as signaling hubs that are activated (or assemble) in response to recognition of pathogenic and cellular products associated with stress and damage. By far, the most well-studied inflammasome is nucleotide-binding domain leucine-rich repeat and pyrin domain-containing receptor 3 (NLRP3). Activation of this inflammasome requires two signals. Signal 1 primes the upregulation of numerous transcripts involved in the inflammasome pathway, including proinflammatory cytokines and NLRP3, while signal 2 promotes the assembly of the inflammasome. NLRP3 oligomerization leads to the recruitment of procaspase-1 via the adapter molecule, apoptosis-associated speck-like protein containing a caspase recruitment domain (ASC; also known as PYCARD). Once in the inflammasome complex, procaspase-1 (p45) undergoes autoproteolysis, generating p33/p10 subunits (Boucher et al., 2018). These active forms of caspase-1 cleave and activate proinflammatory cytokines proIL-1β and proIL-18, as well as gasdermin-D, which forms pores in the plasma membrane, driving cytokine release and cell death by pyroptosis. Caspase-1 undergoes further processing, and p20/p10 dissociates from the inflammasome complex and loses activity (Boucher et al., 2018). Aberrant activation of the NLRP3 inflammasome is linked to various diseases, including diabetes, atherosclerosis, metabolic syndrome, and cardiovascular and neurodegenerative diseases, raising tremendous clinical interest in exploring the potential beneficial/therapeutic effect of NLRP3 inflammasome inhibitors.

Recently, the gut microbiome and its metabolites have received attention as potential causes and/or consequences of obesity (Bakker et al., 2015; Bouter et al., 2017; Coppola et al., 2021; de Clercq et al., 2016; Dugas et al., 2018; Fluitman et al., 2018; Herrema and Nieuwdorp, 2017; Kimura et al., 2020; Koh et al., 2016; Nieuwdorp et al., 2014; Zhang et al., 2020). Seminal work by Bäckhed et al. showed that germ-free mice are protected from developing diet-induced obesity, demonstrating the crucial role played by the gut microbiome in energy balance, substrate utilization and glucose homeostasis (Bäckhed et al., 2004). A novel study by Mocanu et al. revealed that a diet rich in fermentable fibers in conjunction with a single-dose fecal transplantation from lean donors to obese patients improved insulin sensitivity (Mocanu et al., 2021). This work demonstrated that modulation of the gut microbiome holds a therapeutic promise in alleviating obesity-associated comorbidities. The microbiome produces byproducts from fiber fermentation, including short-chain fatty acids: butyrate, propionate and acetate. We and others have shown that a Western diet (WD; 42% kcal from fat, 34% sucrose by weight and 0.2% cholesterol total) led to alterations in gut microbiome composition, decreasing the relative abundance of butyrate producers (Bonomo et al., 2020). Alterations of the gut microbiome, and specifically the decrease in butyrate-producing species, are commonly observed in obesity (Coppola et al., 2021). Butyrate supplementation improves some complications of obesity including glucose intolerance (Coppola et al., 2021; Dalile et al., 2019; Kimura et al., 2020; Koh et al., 2016), heart disease (Trøseid et al., 2020) and pain (Bonomo et al., 2020; Kukkar et al., 2014), but also protects against WD-induced weight gain (Lin et al., 2012). However, no molecular mechanisms underlying butyrate's effects have clearly been identified in obese models. Reports have demonstrated that butyrate has anti-inflammatory effects, regulating T-cell function and macrophage polarization, and can act as an antimicrobial (Arpaia et al., 2013; Chang et al., 2014; Ji et al., 2016; Kimura et al., 2020; Kukkar et al., 2014; Schulthess et al., 2019; Zou et al., 2019). Particularly, butyrate decreases the production of inflammatory cytokines, including IL-1β, in adipose tissue of a genetic mouse model of obesity (*db/db*) (Wang et al., 2015).

In the present work, we took advantage of a recently engineered mouse model to longitudinally monitor caspase-1-mediated inflammation *in vivo* in male and female WD-fed mice. Using this system and secondary confirmatory analyses, we measured caspase-1 activation and associated signaling in WD-fed mice and WD-fed mice treated with tributyrin. We utilized tributyrin – composed of three molecules of butyrate attached to a glycerol backbone – owing to its slower metabolization rate compared to that of sodium butyrate (NaB), which allows circulating butyrate to be absorbed (Gaschott et al., 2001). We focused our measurements on tissues known to be dysregulated in obesity, including adipose tissues, heart, pancreas, liver, intestine and brain. We observed sex- and tissue-specific inflammatory phenotypes upon WD nutrition and butyrate treatment.

## RESULTS

### Caspase-1 biosensor mice display expected WD-induced phenotype

We used the caspase-1 biosensor model that has been recently generated to monitor inflammation *in vivo* in a spatiotemporal manner (Talley et al., 2019). Briefly, transgenic mice express a circularly permuted form of luciferase that becomes bioluminescent in response to active caspase-1, allowing the visualization of caspase-1-mediated inflammatory response in live animals and tissues (Talley et al., 2019, 2021). To validate the use of this model, we first confirmed that the genetic manipulation did not affect whole-body metabolism and response to WD. Biosensor mice were placed on normal chow (NC) (17% kcal from fat, 44.3% carbohydrates by weight) or WD feeding paradigms for a total of 20 weeks to induce long-term obesity-associated complications, including low-grade systemic inflammation (Christ and Latz, 2019; Mansuy-Aubert et al., 2015), and metabolic parameters were assessed longitudinally.

**Fig. 1.**
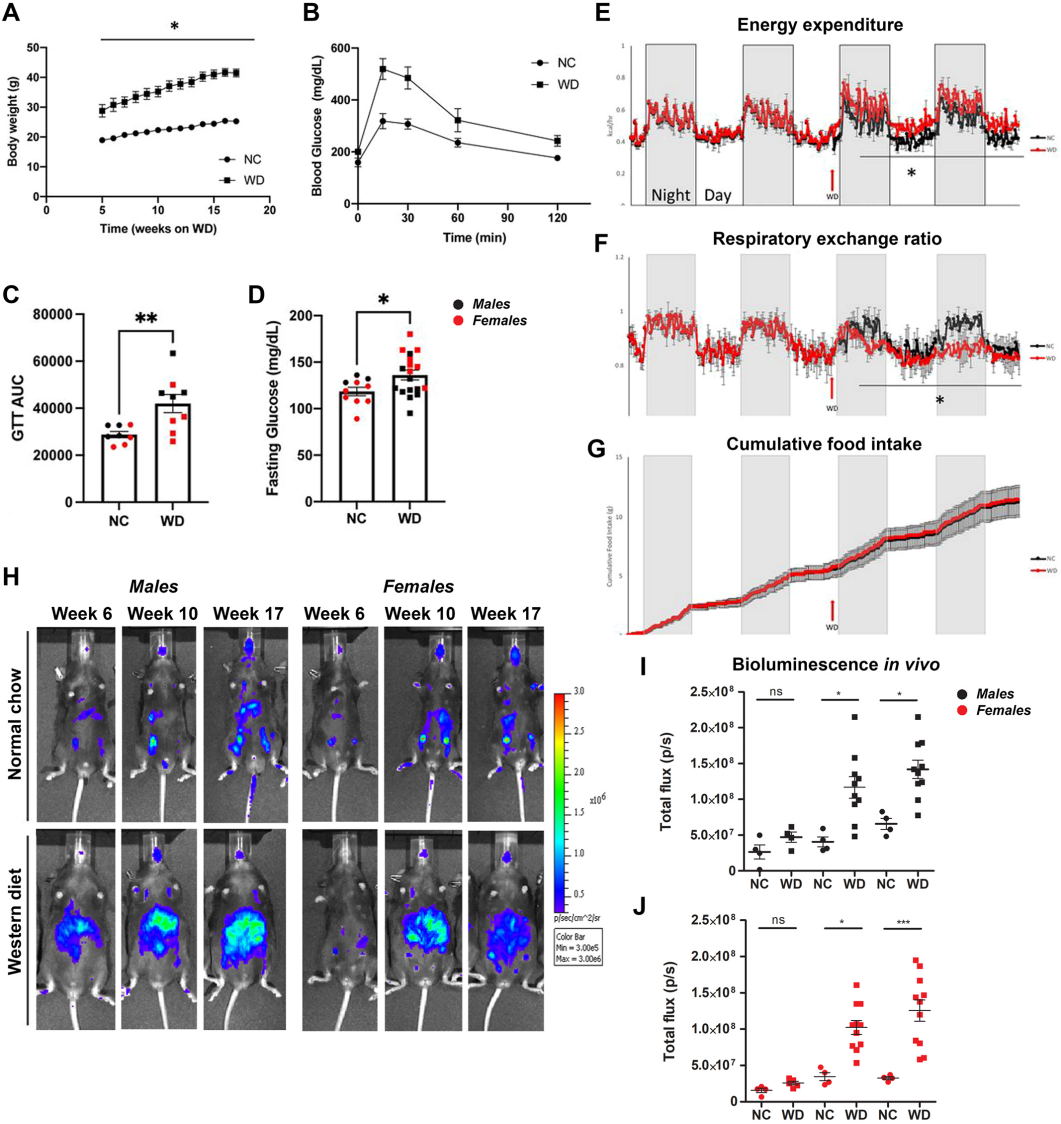
**Obese mice exhibit gradual increase in whole-body caspase-1 activation *in vivo*.** (A) Body weight of normal chow (NC)-fed and Western diet (WD)-fed mice. (B) Glucose tolerance test (GTT) comparison between NC-fed and WD-fed mice. (C) Representative GTT area under the curve (AUC). (D) Overnight fasting blood glucose levels. (E-G) Energy expenditure (E), respiratory exchange ratio (F) and cumulative food intake (G) from NC-fed and WD-fed biosensor mice. (H-J) Representative *in vivo* IVIS images (H) and bioluminescence quantification (I,J) from NC-fed and WD-fed male and female mice. Unpaired two-tailed Student's *t*-test between NC and WD with repeated measures (A); unpaired two-tailed Student's *t*-test between NC and WD (B-G); paired two-tailed Student's *t*-test comparing bioluminescence between NC and WD across the indicated time points (I,J). All values represent mean±s.e.m. ns, not significant; *P<0.05, **P<0.01, ***P<0.001. *n*=4-8 mice per group.

We measured expected WD-induced metabolic syndrome features, such as increased body weight in comparison to NC-fed mice (Fig. 1A), accompanied by WD-induced glucose intolerance (Fig. 1B,C). WD-fed biosensor animals also presented elevated fasting glucose levels (Fig. 1D) after 17 weeks on the feeding paradigm. We also confirmed an increase in energy expenditure (Fig. 1E) and decreased respiratory exchange ratio (Fig. 1F), with no changes in cumulative food consumption (Fig. 1G), when the mice were switched from NC to WD. Our data demonstrate that the caspase-1 biosensor mouse model shows expected behavioral features upon prolonged WD feeding in males and females.

### Obese mice exhibit a gradual increase in whole-body caspase-1 activation *in vivo*

Concurrent with phenotypic analyses, we performed longitudinal imaging of NC- and WD-fed biosensor mice. Males and females were imaged using the *in vivo* imaging system (IVIS). Relative to lean controls, both male and female WD-fed mice showed a rise in bioluminescence signal from week 6 to week 10 that was sustained to week 17 of the WD feeding paradigm (Fig. 1H-J). Together, our results indicate that the WD led to the activation of the engineered caspase-1 biosensor in male and female mice.

### WD and tributyrin alter caspase-1 activation, inflammatory transcript expression and cytokine expression differentially in tissues from male and female mice

**Fig. 2.**
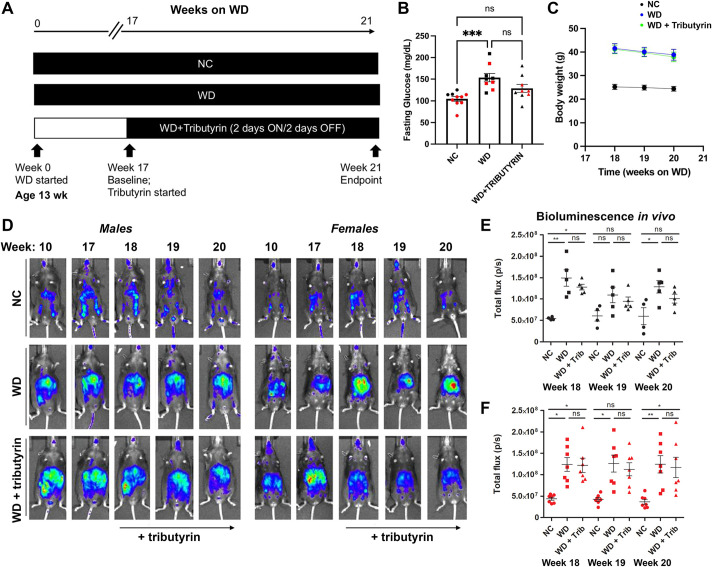
**Short duration of tributyrin treatment does not affect fasting glucose, body weight or caspase-1 biosensor activation in obese mice.** (A) Experimental paradigm. (B) Baseline overnight fasting glucose in NC-fed, WD-fed and WD-fed tributyrin-treated male and female mice. (C) Body weight of NC-fed, WD-fed and WD-fed tributyrin-treated mice during the 2 weeks of tributyrin treatment. (D-F) Representative *in vivo* IVIS images (D) and bioluminescence quantification (E,F) from NC-fed, WD-fed and WD-fed tributyrin-treated male and female mice. One-way ANOVA and Tukey's post-hoc test. All values represent mean±s.e.m. ns, not significant; *P<0.05, **P<0.01, ***P<0.001. *n*=4-8 mice/group for males and females.

Numerous studies have demonstrated that long-term butyrate treatment is protective in mouse models of diet-induced obesity, reducing body weight gain, insulin resistance or cardiovascular events in obese mice (den Besten et al., 2015; Gao et al., 2009) and attenuating obesity-associated inflammation. To better understand the early mechanistic changes driving these protective effects, we aimed to develop a paradigm of short-term tributyrin administration that would allow us to assess the potential inflammatory changes occurring early in obese mice prior to butyrate-induced weight loss and improved fasting glucose and cardiovascular effects. First, we defined the dose and time course of butyrate administration. In a dose-response pilot study, using serum lipidomics, we observed that 5 g/kg of tributyrin led to a 25-fold increase in circulating butyrate after 2 h, persisting until 48 h post-treatment. At the end of week 17 of WD feeding, when mice exhibited high levels of caspase-1 activation *in vivo*, we established a treatment paradigm by administrating 5 g/kg tributyrin every 48 h (2 days on/2 days off) to the biosensor mouse model for 2 weeks (Fig. 2A). Two weeks of tributyrin trended towards attenuating fasting glucose levels of WD-fed mice (Fig. 2B) without an effect on body weight (Fig. 2C). This duration of tributyrin administration did not significantly reduce whole-body biosensor activation *in vivo* in obese male and female mice (Fig. 2D-F).

**Fig. 3.**
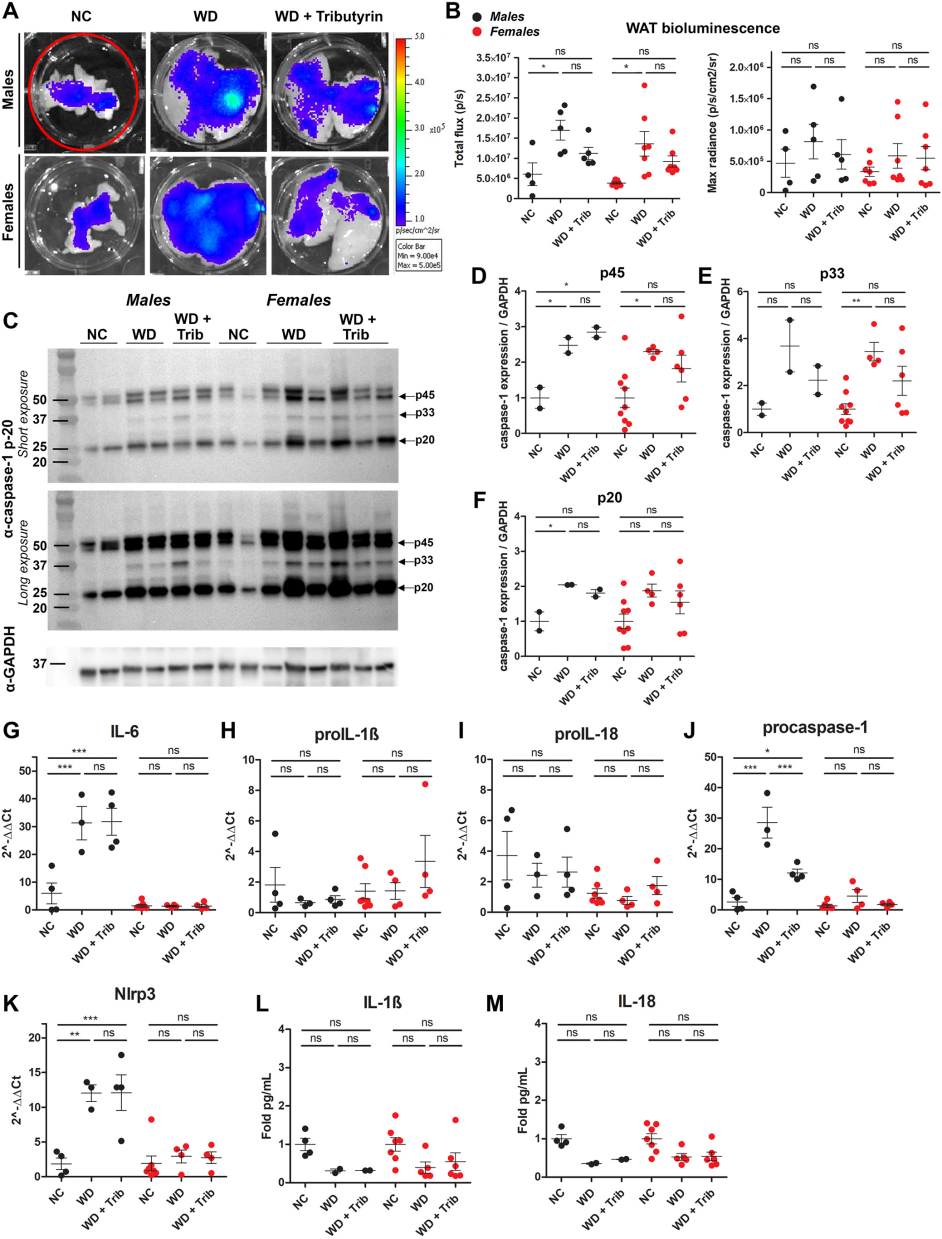
**WD increases caspase-1 activation in the white adipose tissue (WAT).** (A,B) Representative *ex vivo* IVIS images (A) and bioluminescence quantification (B) of WAT from NC-fed, WD-fed and WD-fed tributyrin-treated male and female mice. (C-F) Caspase-1 protein expression in the WAT was measured by western blotting (C), and the indicated bands [p45 (D), p33 (E) and p20 (F)] were quantified using ImageJ. (G-K) RNA isolated from the WAT was converted to cDNA, and the expression of proinflammatory transcripts was measured by qRT-PCR. (L,M) IL-1β (L) and IL-18 (M) cytokine levels were measured by ELISA. One-way ANOVA and Tukey's post-hoc test. All values represent mean±s.e.m. ns, not significant; *P<0.05, **P<0.01, ***P<0.001. *n*=2-8 mice/group for males and females.

At endpoint (20 weeks WD), we investigated the tissue-specific effects of NC, WD and tributyrin on caspase-1 activation as measured by tissue bioluminescence and western blotting. We also completed our analyses with the measurement of inflammatory transcripts and cytokine expression (Table 1). We aimed at evaluating caspase-1 activation in metabolic tissues including white and brown adipose tissue (WAT and BAT, respectively), heart, liver, pancreas, intestine and brain. We hypothesized that the predominant source of the biosensor signal measured *in vivo* in WD-fed male and female mice was primarily the adipose tissue surrounding the peritoneal cavity, including subcutaneous, gonadal and mesenteric fat depots (Fig. 1H and Fig. 2D). Consistent with the *in vivo* data, we measured a significant increase in total flux (p/s) of bioluminescence signal in the WAT between NC- and WD-fed male and female mice, likely due to the increase in total abdominal fat (Fig. 3A,B). However, because WAT size varied among animals belonging to the different experimental groups, we also analyzed the maximum radiance emitted by the tissue (p/s/cm^2^/sr), which is irrespective of tissue size. We did not detect changes in WAT maximum radiance among the experimental groups in either males or females (Fig. 3B).

**Table 1.**
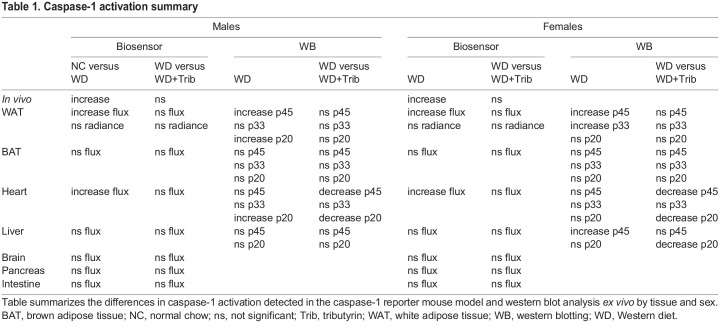
Caspase-1 activation summary

To confirm and further characterize the inflammatory responses activated in this tissue in WD-fed mice, we measured caspase-1 activation by western blotting, proinflammatory transcript expression by quantitative real-time PCR (qRT-PCR), and IL-1β and IL-18 cytokine levels by enzyme-linked immunosorbent assay (ELISA). WD-fed mice exhibited a significant increase in procaspase-1 (p45) and cleaved caspase-1 (p33 and p20), which was not significantly reduced by short-term tributyrin administration (Fig. 3C-F). We also measured an upregulation of the proinflammatory transcripts *Il6*, procaspase-1 and *Nlrp3* in WD-fed male mice adipose, but none of these transcripts were upregulated in WD-fed females (Fig. 3G-K). Notably, tributyrin significantly reduced procaspase-1 expression in WD-fed male mice (Fig. 3J). Despite this increased proinflammatory signature in the WAT of WD-fed mice, we did not detect transcriptional changes or increased protein expression for IL-1β and IL-18 (Fig. 3H-I,L,M). Together, these data indicate that caspase-1 activation and the expression of some inflammatory transcripts were increased in the WAT of WD-fed male and female mice.

**Fig. 4.**
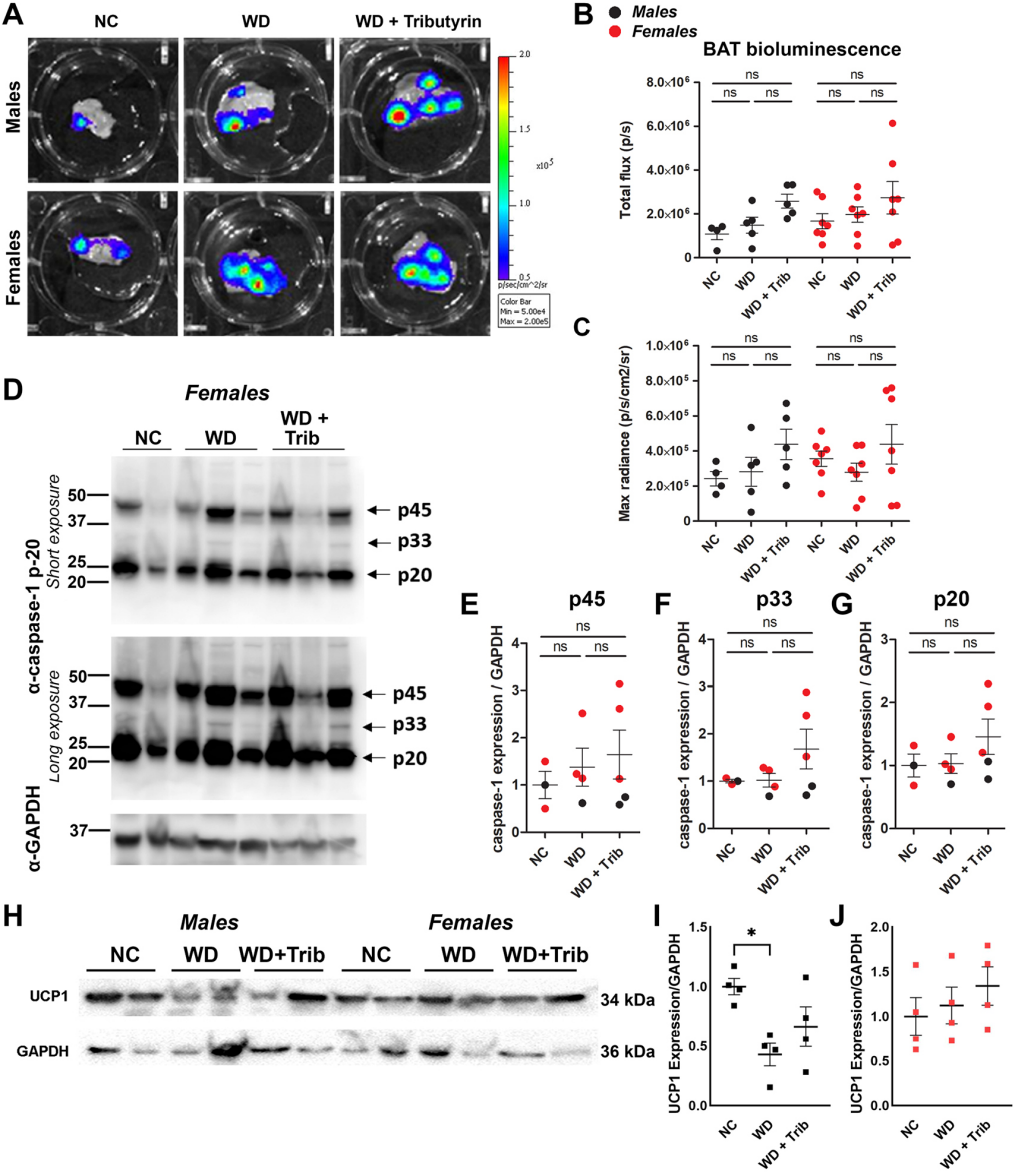
**WD does not significantly increase caspase-1 activation in the brown adipose tissue (BAT).** (A-C) Representative *ex vivo* IVIS images (A) and bioluminescence quantification (B,C) of the BAT from NC-fed, WD-fed and WD-fed tributyrin-treated male and female mice. (D-G) Caspase-1 protein expression in the BAT was measured by western blotting (D), and the indicated bands [p45 (E), p33 (F) and p20 (G)] were quantified using ImageJ. (H-J) UCP1 protein expression in the BAT was measured by western blotting (H) and quantified using ImageJ (I,J) One-way ANOVA and Tukey's post-hoc test. All values represent mean±s.e.m. ns, not significant; *P<0.05. *n*=3-7 per group.

We next evaluated caspase-1 activation in the BAT together with the use of the well-defined BAT marker uncoupling-protein 1 (UCP1). In contrast to WAT, we did not detect significant differences in caspase-1 activation in the BAT, although there was a trend toward increased bioluminescence and cleaved caspase-1 expression in tributyrin-treated WD-fed mice (Fig. 4A-G). In rodent models of obesity, thermogenesis is impaired due to low UCP1 expression (Alcalá et al., 2019). We detected reduced UCP1 protein levels in the BAT of WD-fed male mice, and UCP1 levels were unaltered by the tributyrin treatment paradigm (Fig. 4H-J).

**Fig. 5.**
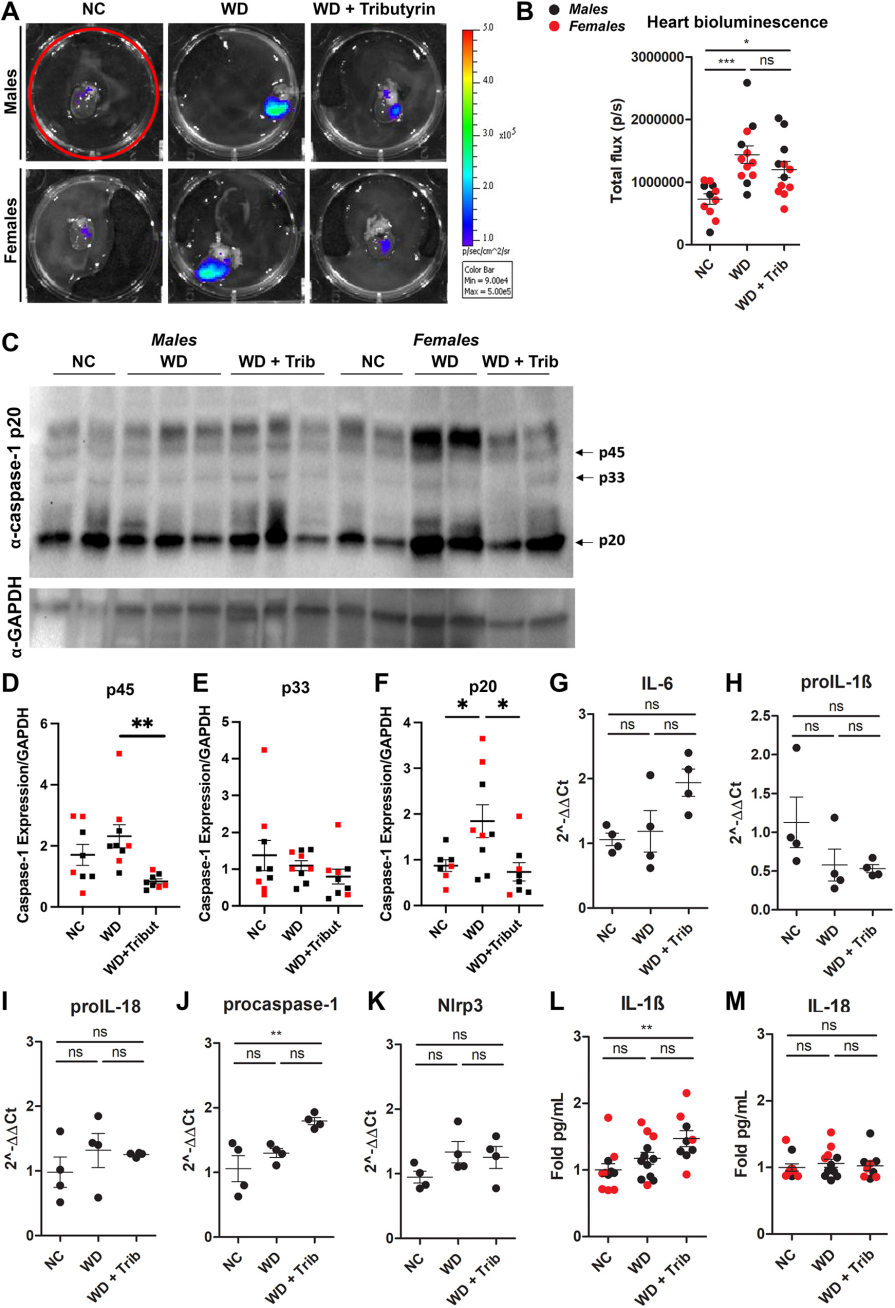
**WD increases some markers of inflammation in the liver.** (A,B) Representative *ex vivo* IVIS images (A) and bioluminescence quantification (B) of liver tissue from NC-fed, WD-fed and WD-fed tributyrin-treated male and female mice. (C-F) Caspase-1 protein expression in the liver was measured by western blotting (C), and the indicated bands [p45 (D), p33 (E) and p20 (F)] were quantified using ImageJ. (G-K) RNA isolated from the liver was converted to cDNA and expression of proinflammatory transcripts was measured by qRT-PCR. (L,M) IL-1β (L) and IL-18 (M) cytokine levels were measured by ELISA. One-way ANOVA and Tukey's post-hoc test. All values represent mean±s.e.m. ns, not significant; *P<0.05, **P<0.01, ***P<0.001. *n*=2-8 mice/group for males and females.

We then measured caspase-1 activation in the hearts of animals from the aforementioned groups. We detected a significant increase in caspase-1 activation, measured by tissue bioluminescence, in both males and females (Fig. 5A,B). To validate our data, we performed western blot analysis of heart tissue from NC-fed, WD-fed and WD-fed tributyrin-treated mice. In agreement with the biosensor data, we measured increased activation of caspase-1 in heart tissue from obese mice compared to that from lean controls, as measured by a significant increase in cleaved caspase-1 (p20) (Fig. 5C,F). We also detected an attenuation of caspase-1 activation by tributyrin treatment, as observed by a significant decrease in procaspase-1 (p45) and cleaved caspase-1 (p20) expression (Fig. 5C-F). Despite this increase in caspase-1 activation, we did not measure significant changes in proinflammatory transcript expression and cytokine levels in heart tissue from WD-fed mice (Fig. 5G-M).

**Fig. 6.**
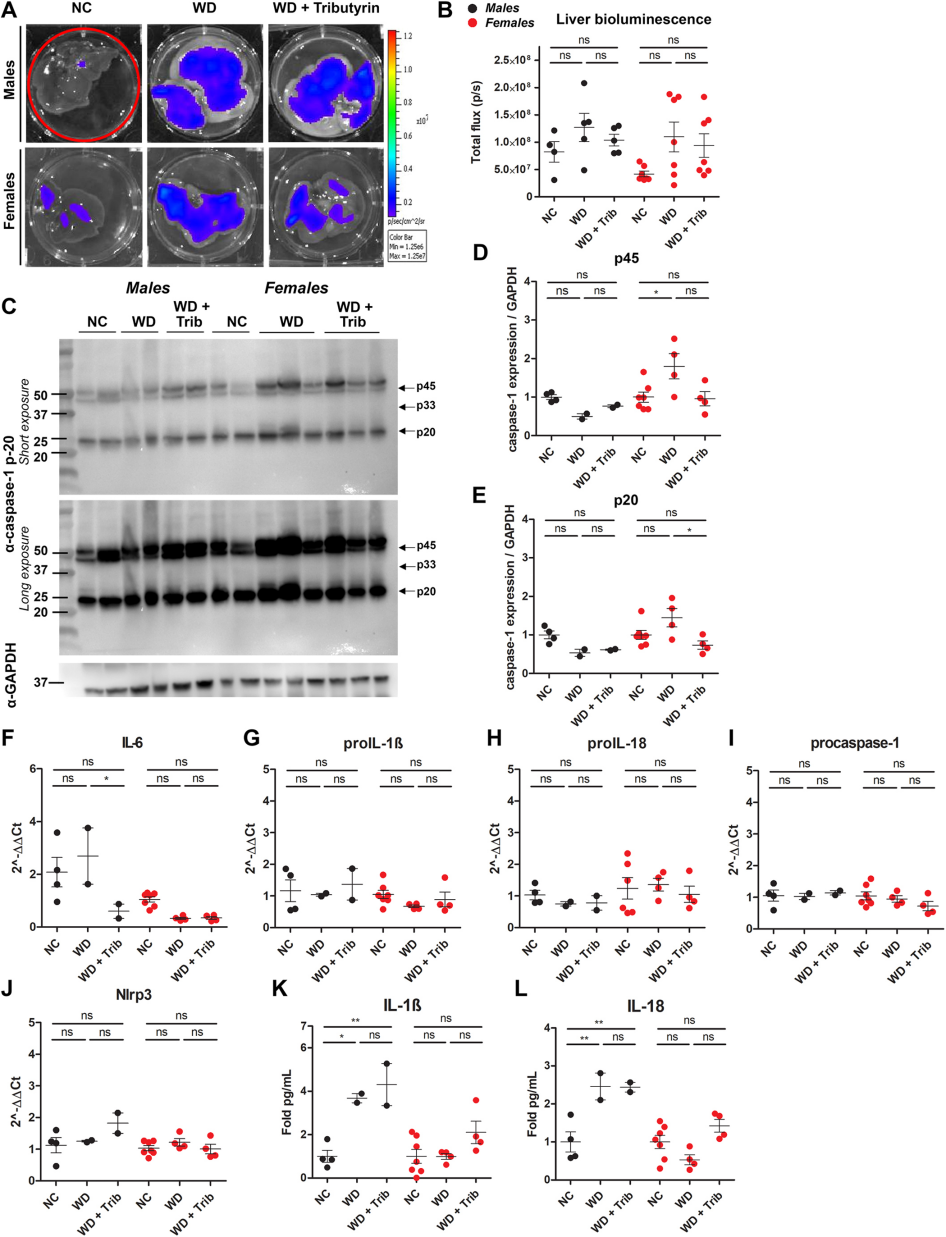
**WD increases caspase-1 activation in the heart.** (A,B) Representative *ex vivo* IVIS images (A) and bioluminescence quantification (B) of heart tissue from NC-fed, WD-fed and WD-fed tributyrin-treated male and female mice. (C-E) Caspase-1 protein expression in the heart was measured by western blotting (C), and the indicated bands [p45 (D) and p20 (E)] were quantified using ImageJ. (F-J) RNA isolated from the heart of male mice was converted to cDNA, and expression of proinflammatory transcripts was measured by qRT-PCR. (K,L) IL-1β (K) and IL-18 (L) cytokine levels were measured by ELISA. One-way ANOVA and Tukey's post-hoc test. All values represent mean±s.e.m. ns, not significant; *P<0.05, **P<0.01, ***P<0.001. *n*=2-8 mice/group for males and females.

Subsequently, we analyzed biosensor activation in livers from NC-fed, WD-fed and WD-fed tributyrin-treated male and female mice. We did not detect a significant increase in total flux in the livers from obese male mice compared to those from lean controls, and total flux was also unaltered by tributyrin treatment (Fig. 6A,B). In female mice, there was a trend toward increased bioluminescence in the livers of WD-fed mice and a measurable increase in caspase-1 expression (p45) (Fig. 6A-D). Expression of cleaved caspase-1 (p20) was reduced in tributyrin-treated female mice (Fig. 6E). Although we did not measure any significant differences in proinflammatory transcript expression (Fig. 6F-J), IL-1β and IL-18 protein levels were notably higher in liver tissue from WD-fed male mice (Fig. 6K,L) .

Finally, we did not detect significant changes in tissue bioluminescence signal from brain, pancreas and intestine in either male or female mice (Fig. S1A-F).

### Butyrate stimulation modifies expression of proinflammatory transcripts in bone marrow-derived macrophages (BMDMs) from obese mice

**Fig. 7.**
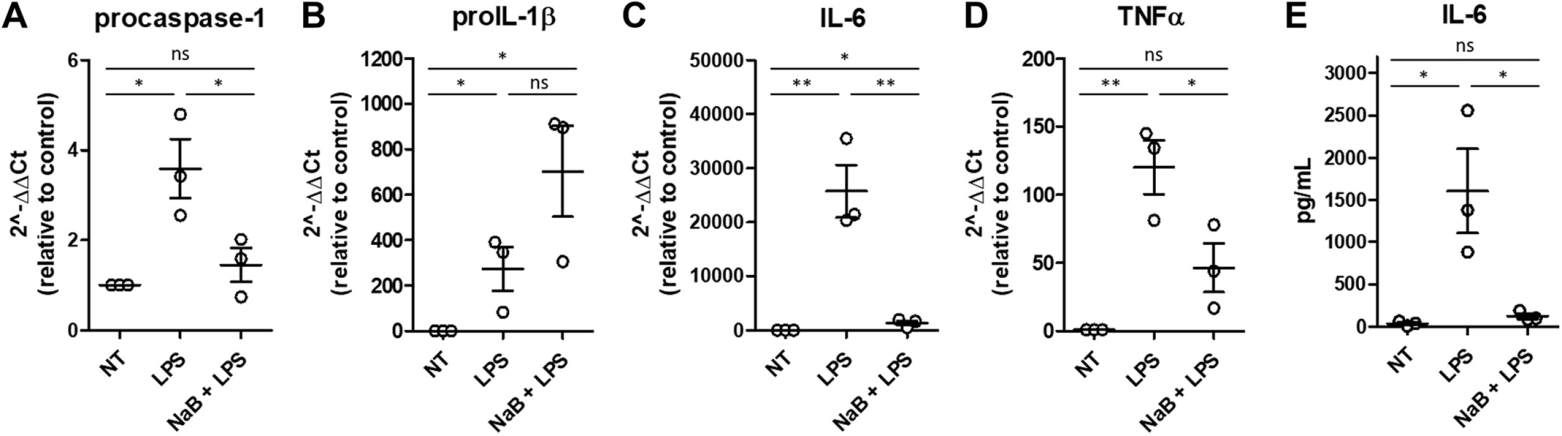
**Butyrate modifies cytokine expression in bone marrow-derived macrophages (BMDMs).** BMDMs derived from WD-fed mice were stimulated with LPS or LPS+sodium butyrate (NaB). (A-E) Inflammatory transcript expression was quantified by qRT-PCR (A-D), and cytokine secretion was measured by cytokine bead array (E). One-way ANOVA and Tukey's post-hoc. All values represent mean±s.e.m. ns, not significant; *P<0.05, **P<0.01. *N*=3 independent experiments; *n*=3 male mice/group.

Infiltration of inflammatory macrophages and other immune cells triggers a proinflammatory environment in WAT and other tissues in WD-induced obese mice. Because the caspase-1 reporter is expressed in all cells and tissues, we were unable to determine the source of biosensor signal and caspase-1 activation in mice and in affected tissues. To test the hypothesis that the changes in caspase-1 activation observed in adipose tissue and heart from WD-fed mice could be due to an effect on macrophages, we stimulated BMDMs from WD-fed mice with lipopolysaccharide (LPS) in the presence or absence of NaB. Exposure to NaB significantly decreased LPS-induced mRNA expression of procaspase-1, *Il6* and *Tnfa* (also known as *Tnf*) (Fig. 7A,C,D) as well as IL-6 secretion (Fig. 7E). proIL-1β mRNA expression was significantly higher in LPS-stimulated BMDMs compared to non-treated macrophages, and NaB exposure did not reduce the expression of this proinflammatory transcript (Fig. 7B). Collectively, these results suggest that butyrate may have a protective role in preventing LPS-induced activation of inflammatory responses in macrophages in obese mice, partially by decreasing the expression and production of inflammatory cytokines

### Pathway analysis based on published databases: FFAR2-mediated pathway, when stimulated by butyrate, suppresses the LPS-mediated classical activation of macrophages

**Fig. 8.**
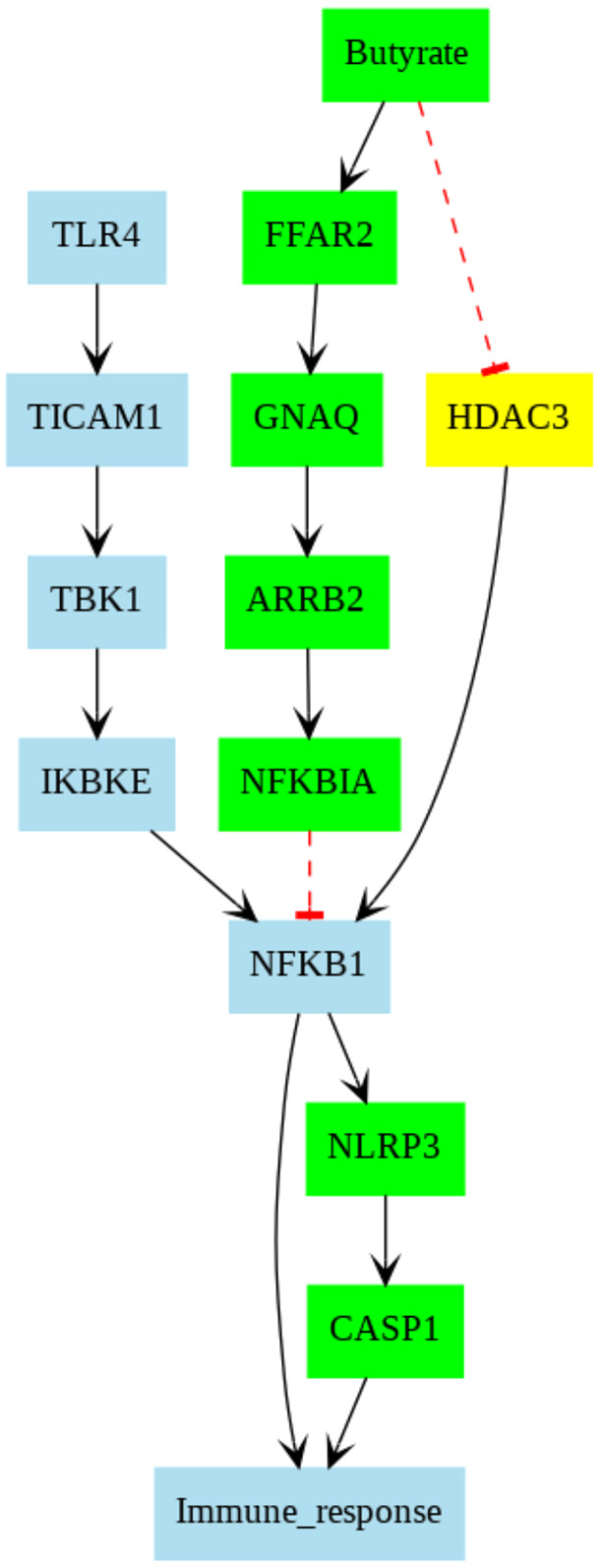
**Pathways collected from STRING database.** Each color corresponds to a specific pathway. Black- and red-colored pathways are from LPS/TLR4- and FFAR2-mediated pathways, respectively. The black arrows indicate promoting correlation, whereas red dashed lines with blunt ends denote inhibitory correlation.

Given our hypothesis that changes in caspase-1 activity due to butyrate were mediated by macrophages, we investigated the putative proinflammatory pathways that might be suppressed by short-chain fatty acids. We performed a computational analysis of a manually curated signaling network for macrophages based on the following databases: Kyoto Encyclopedia of Genes and Genomes (KEGG), STRING and SIGNOR (Fig. 8). Our procedure consisted of four steps: (1) identification of LPS-mediated pathways culminating in caspase activity, (2) a query for genes susceptible to butyrate, (3) identifying butyrate-mediated genes that are linked to the LPS-mediated pathways using protein–protein interaction pairs (PPIs) identified from PPI database, and (4) traversing the pathways to identify those suppressing caspase-1 activity. Details of this approach, including the database and the network traversal algorithm, are described in the Materials and Methods section.

Our curated network reflects that classical M1 activation via LPS/TLR4 complex promotes the activation of NFκB p65/p50 (associated gene, *Nfkb1*), which initiates gene transcription associated with proinflammatory cytokines or inflammatory species, such as IL-1β or NLRP3/caspase-1 complex (Licata et al., 2020; Paik et al., 2021), respectively. Our pathway analysis based on the published databases suggests that the free fatty acid receptor 2 (FFAR2)-mediated pathway, when stimulated by butyrate, suppresses the LPS-mediated classical activation of macrophages. This occurs through butyrate stimulation of the G-protein-coupled receptor FFAR2 (He et al., 2020), which promotes β-arrestin 2 (associated gene, *Arrb2*) activity and ultimately inhibition of the NFκB complex. ARRB2 has previously been established to suppress NFκB activation via promoting its inhibition by NFκB inhibitor alpha (associated gene, *Nfkbia*) (Szklarczyk et al., 2019; Witherow et al., 2004). Hence, reduction of caspase-1 in tributyrin-treated WD-mice is expected and may stem from FFAR2-mediated suppression of NFκB activity. The analysis also indicated that butyrate may directly inhibit HDAC3 activity (Walsh and Van Remmen, 2016), which also results in suppression of transcription of proinflammatory transcripts via NFκB activation.

## DISCUSSION

Herein, we investigated the effects of WD feeding and tributyrin treatment on whole-body and tissue-specific inflammation. Using a method that allowed for monitoring of caspase-1-mediated inflammation longitudinally *in vivo*, we found that WD feeding led to a gradual increase in caspase-1 activation over time. The source of the bioluminescence signal originated from the abdominal cavity, which implied that this increase in inflammation measured in obese mice could have occurred in (1) WAT (subcutaneous and visceral fat), (2) internal organs, including heart, liver, pancreas and intestines, or (3) the peritoneal cavity – loaded with immune cells, such as macrophages.

To confirm the source of increased caspase-1 activation measured *in vivo*, we analyzed caspase-1 activation and inflammatory responses in tissues *ex vivo* at the study endpoint. Our approach included measuring caspase biosensor activation *ex vivo* in tissues as well as changes in caspase-1 expression and activation by western blotting. In most tissues, we were able to measure expression of three bands, corresponding to ∼p45, p32-p33 and p20. The increased expression of p33 and p20 is indicative of caspase-1 autoproteolysis, demonstrating that caspase-1 is or has been active in an inflammasome complex, whereas p45 can correspond to procaspase-1 or an active species (Boucher et al., 2018). We also quantified inflammatory transcript expression, which is a measurement of ‘signal 1’ transcriptional priming for inflammasome activation, and we quantified the expression of IL-1β and IL-18, two cytokines processed by active caspase-1. Critically, the process of events of inflammatory transcript upregulation, inflammasome formation, caspase-1 activation, cytokine processing and biosensor activation would likely be temporally separated in cells and between different cell types within tissues. Therefore, we would not expect a perfect correlation between all measurements of inflammation in inflamed tissues, and this would be especially true in a chronic inflammatory disease. This underlies the key difficulty in measuring inflammatory responses, which are inherently dynamic, at a static endpoint *ex vivo*.

We detected significant differences in bioluminescent signal in WAT and heart tissue that could be responsible, at least in part, for the increase in inflammation observed *in vivo*. Our model, however, lacks cell specificity, and further analyses would be necessary to identify which cells are responsible for caspase-1 activation within these tissues. For instance, WAT is not only composed of adipocytes, but it is also richly innervated by autonomic nerve fibers and is a main location for specialized immune cells (Pirzgalska et al., 2017; Ruggiero et al., 2021). WAT expansion occurs during obesity, concomitant with enlargement of old and newly formed adipocytes (Ruggiero et al., 2021). During WAT expansion, this highly specialized tissue becomes infiltrated with a plethora of immune cells, contributing to the systemic low-grade inflammatory status of obesity (Ruggiero et al., 2021). Different subtypes of macrophages inundate WAT, but proinflammatory M1 macrophages are thought to be the dominant polarized state (Ruggiero et al., 2021). These cells are known to produce inflammatory cytokines, such as IL-6 and IL-1β, and, when in excess, they can lead to tissue damage and disruption of tissue function (Ruggiero et al., 2021). Sympathetic-associated macrophages (SAMs) are a newly identified macrophage subtype that lies in close association with sympathetic nerve fibers within WAT (Pirzgalska et al., 2017). SAMs have been described to play a fundamental role in obesity by mediating norepinephrine uptake in WAT (Pirzgalska et al., 2017). Although they share features with M1 macrophages, SAMs have a unique phenotype by mediating noradrenergic-induced WAT lipolysis and reduction of fat mass (Pirzgalska et al., 2017). Moreover, these cells have been previously demonstrated to increase IL-1 expression in obese mice adipose tissue (Pirzgalska et al., 2017). M1 macrophages or SAMs could be the cells mediating the increase in caspase-1 activation in our model, because we not only observed a significant increase in caspase-1 activation in the whole WAT, but we also measured a significant increase in the expression and production of inflammatory transcripts in LPS-stimulated BMDMs isolated from WD-fed mice *in vitro*.

In addition to WAT, we measured a significant increase in caspase-1 biosensor activation in heart tissue from WD-fed mice relative to that from NC-fed control mice (Fig. 5A,B). Consistent with these data, cleaved caspase-1 (p20) expression was also increased in the hearts from WD-fed mice and tributyrin attenuated caspase-1 p45 and p20 expression (Fig. 5C-F). Obese individuals are at higher risk for developing cardiovascular disease, and systemic low-grade inflammation is known to impair cardiac remodeling (Mouton et al., 2020). Immune cell infiltration and M1 macrophage polarization, both associated with obesity and metabolic syndrome components, lead to cardiac injury (Mouton et al., 2020). In addition, gut microbiome alteration in obesity has recently been linked to cardiovascular disease and myocardial infarct (Battson et al., 2019). Obese mice transplanted with cecal contents from lean animals presented reduced myocardial infarct size, concomitant with alterations in gut permeability and cecal butyrate content (Battson et al., 2019). Dysregulation of the gut microbiota has also been linked to the development of coronary artery disease (CAD) and heart failure (HF) (Trøseid et al., 2020). Remarkably, the literature suggests that imbalance of circulating levels of microbiome-derived metabolites, alongside a decrease in butyrate producers, are potential biomarkers for CAD and HF (Trøseid et al., 2020). More detailed studies can provide deeper knowledge on the more specific roles of WAT and heart in inflammation in obesity and how tributyrin can affect caspase-1 activation.

The main function of the BAT is thermogenesis – to produce heat by dissipating energy. Uncoupling proteins (UCPs) are the drivers of BAT thermogenesis, as they dissipate mitochondrial proton gradient and allow the uncoupling between the respiratory chain and ATP generation (Alcalá et al., 2019). In our WD model (42% fat), we did not observe changes in biosensor signal in BAT from WD-fed compared to NC-fed mice. In contrast to the WAT, we also did not detect any increase in active caspase-1 or inflammatory cytokines in the BAT. These data are consistent with previous observations that BAT is less susceptible to WD-induced inflammation/inflammatory cell infiltration compared to WAT (Li et al., 2018; Keuper and Jastroch, 2021; Palmer and Clegg, 2015). However, thermogenesis was impaired in the obese male mice, as measured by reduced UCP-1 expression (Fig. 5H,I). There was also a trend toward increased UCP-1 expression in the tributyrin-treated mice (Fig. 5I). This is supported by previous work demonstrating that oral butyrate treatment led to increased BAT thermogenic capacity (Li et al., 2018). Although female rodents challenged with high-fat diets are known to have higher expression of thermogenic genes (Keuper and Jastroch, 2021), we did not detect differences in UCP1 protein levels in our model (Fig. 5J). This inconsistency in results could be due to variations in diet composition and length and should be further investigated.

We also did not measure significant WD-induced inflammatory phenotypes in the pancreas, intestine or brain. We cannot rule out the possibility that caspase-1 is active in these tissues earlier or later in the WD-induced obesity model and that we may have missed these measurements at the time of our study endpoint.

Previous studies have reported on the sexual dimorphism of obesity (Palmer and Clegg, 2015). Differential adipose tissue distribution and function between men and women that lead to distinct metabolic and neural features are mainly driven by the hormonal patterns in both sexes (Palmer and Clegg, 2015). Some of the results presented highlight WD-associated sex differences in inflammation. For example, the inflammatory transcripts *Il6*, procaspase-1 and *Nlrp3* were specifically upregulated in WAT in WD-fed males (Fig. 3G,J,K), and IL-1β and IL-18 levels were significantly upregulated in the liver of WD-fed males (Fig. 6K,L), but none of these inflammatory pathways were upregulated in females. This suggests a differential regulation of inflammatory pathways between males and females upon WD feeding. It also agrees with the literature highlighting that females are more resistant to WD than males, and they develop earlier complications such as insulin resistance likely associated with inflammation (Elzinga et al., 2021). Estrogen (E2) signaling is known to contribute to reduction of adipose tissue inflammation through activation of estrogen receptor alpha (ERa), which leads to regulation of hypoxia-inducible factor (HIF) (Palmer and Clegg, 2015). In obesity, the WAT becomes hypoxic, and HIF-1 is upregulated. This, in turn, increases production of inflammatory mediators, including IL-6 (Palmer and Clegg, 2015). It is plausible that the differential inflammatory response observed in females in our model could be due to E2/ERa signaling; however, future studies should investigate these differences in inflammation between males and females upon WD feeding and butyrate treatment and the potential role of sex hormones.

Lastly, our *in vitro* data revealed that NaB exposure decreased the expression of proinflammatory transcripts, procaspase-1, *Il6* and *Tnfa*, and reduced secretion of the proinflammatory cytokine IL-6 in LPS-stimulated BMDMs isolated from WD-fed mice (Fig. 7). These results indicate that acute butyrate exposure confers protection against LPS-induced proinflammatory responses in cultured BMDMs. Consistent with these data, we also measured reduced caspase-1 expression in the WAT of tributyrin-treated WD-fed male mice (Fig. 3J). This suggests that macrophages could be contributing to inflammasome/caspase-1 activation in the WAT and heart, and downregulation of inflammatory transcripts in these cells, specifically caspase-1, which may be responsible for the protective, anti-inflammatory effects of long-term butyrate administration in obese mice. Importantly, although macrophages and other immune cells are known to trigger a proinflammatory environment, caspase-1 activation occurs in numerous cell types, which can confound interpretation. A cell-specific bioengineered mouse model would be a more relevant tool to better determine which cell types are driving inflammation *in vivo* and in specific tissues during obesity and which cell types are responsive to butyrate.

Our work demonstrated that WD feeding led to a gradual increase in caspase-1 activation *in vivo* over time, indicating the potential utility of this caspase-1 reporter mouse model for measuring the onset of inflammation in models of obesity and other chronic inflammatory diseases. Although we identified upregulation of inflammatory pathways in the WAT and heart in WD-fed mice, future studies are needed to identify the cells and inflammasome(s) driving these responses during obesity. Our study also assessed changes in caspase-1 activation following a short tributyrin therapeutic intervention, but future work should measure the long-term effects of tributyrin administration on the resolution of caspase-1-dependent inflammatory responses. When combined with other assays to measure inflammation, this caspase-1 biosensor mouse model can be extremely useful to perform tissue-specific drug discovery that would improve complications of obesity impacting specific tissues.

## MATERIALS AND METHODS

### Animal studies

Animal studies were conducted in accordance with recommendations in the Guide for the Care and Use of Laboratory Animals of the National Institutes of Health and with the approval of the Loyola University Chicago Institutional Animal Care and Use Committee. Wild-type, male C57BL/6J (#000664) mice were obtained from The Jackson Laboratory (Bar Harbor, ME, USA). Caspase-1 biosensor mice were generated in-house as described elsewhere (Talley et al., 2019). Briefly, these mice express a circularly permuted luciferase construct that becomes bioluminescent upon proteolytic cleavage by caspase-1, allowing for monitoring of the spatiotemporal dynamics of inflammasome/caspase-1 activation in living mice and in tissues (Talley et al., 2019).

Animals were housed under a 12:12 h light/dark cycle. The lean group was fed NC (Teklad LM-485, Envigo, Indianapolis, IN, USA; 17% kcal from fat, 44.3% carbohydrates by weight), while the experimental groups were fed WD (TD88137, Teklad Diets, Envigo; 42% kcal from fat, 34% sucrose by weight and 0.2% cholesterol total) for 20 weeks starting at 13 weeks of age.

### Tributyrin treatment

Thirteen-week-old mice were fed NC or WD for 20 weeks. At the end of week 17, animals were needle fed with tributyrin (three butyrate molecules attached to a glycerol backbone; 5 g/kg body weight; Sigma-Aldrich) or 1× PBS (used as vehicle) every 48 h (2 days on/2 days off) for 2 weeks (*n*=4-8 mice per group).

### Fasting glucose

Mice were overnight fasted, and glucose was measured from tail blood drops using an AlphaTrak glucometer for rodents (Fisher Scientific, Hampton, NH, USA).

### IVIS imaging

*In vivo* imaging was done as previously described (Talley et al., 2019). Briefly, mice were anesthetized, weighed and injected intraperitoneally with a single dose of 150 mg/kg VivoGlo Luciferin (Promega). Anesthetized mice were imaged with an IVIS 100 Imaging System (Xenogen) 10 min after administration of the luciferase substrate. For *ex vivo* imaging, mice were sacrificed immediately following *in vivo* image acquisition and dissected tissues were placed in a six-well plate with 1 ml luciferase substrate (300 μg/ml). Bioluminescence was quantified and analyzed use Living Image software (Perkin Elmer).

### Western blot analysis

Proteins from frozen mouse hearts were prepared by lysis in ice-cold RIPA buffer (89900, ThermoFisher Scientific) containing protease and phosphatase inhibitor (A32959, ThermoFisher Scientific). Tissue was homogenized using a bullet blender bead lysis kit (Next Advance), and protein concentrations were determined with a Pierce BCA Protein Assay Kit (23225, ThermoFisher Scientific). Proteins were separated by sodium dodecyl sulfate polyacrylamide gel electrophoresis (SDS-PAGE) on 4-15% gradient gel (4561086, Bio-Rad) and transferred to PVDF membrane using an iBlot 2 transfer system (ThermoFisher Scientific). Protein expression was measured by chemiluminescence using a ChemiDoc imaging system (Bio-Rad). Proteins were detected with the following primary antibodies: anti-caspase-1(p20) (AdipoGen; 1:500), anti-β-actin (ab8226, Abcam; 1:1000) and anti-GAPDH (sc-365062, Santa Cruz Biotechnology; 1:1000).

### Culture and treatment of BMDMs

Bone marrow cells were isolated from the femurs and tibias of WD-fed mice and cultured in medium containing 20 ng/ml macrophage colony stimulation factor (M-CSF), as previously described (Dreier et al., 2018; Talley et al., 2019). After 6 days in culture, differentiated BMDMs were re-plated in 24-well plates and treated with 100 ng/ml LPS (from *Escherichia coli* O26:B6; Sigma-Aldrich) with or without 1 mM NaB for 4 h.

### ELISA and cytokine bead arrays (CBAs)

For cytokine analysis in tissues, tissues were processed as described above, and homogenates were centrifuged and lysate collected. Mouse IL-1β and IL-18 ELISAs were performed on tissue homogenates following the manufacturer's instructions (R&D Systems). For *in vitro* experiments, supernatant was collected from BMDMs following treatments. CBA was performed using BD CBA Flex sets for the indicated cytokines (BD Biosciences) following the manufacturer's instructions, and data were analyzed on an LSRFortessa (BD Biosciences).

### Quantitative PCR

Following treatment, BMDMs were washed once with 1× PBS (Corning), and RNA was isolated using an RNEasy Mini Kit (Qiagen). To obtain cDNA, a reverse transcription reaction was performed using a Taq polymerase reverse transcription kit (Applied Biosciences). For all genes of interest (listed in Table 2), quantitative PCR was performed using SYBR Green I-based assay (Roche, Indianapolis, IN, USA) using IDT primers (Integrated DNA Technologies, Coralville, IA, USA). Actin was used to normalize data, and quantification was done using the ΔΔCT method with the vehicle-treated group's mean value set at 100%, as reported previously (Gavini et al., 2018).

**Table 2.**
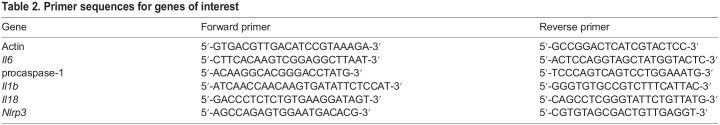
Primer sequences for genes of interest

### Pathway analysis

The manually curated network was built from several databases including KEGG, STRING and SIGNOR (Kanehisa and Goto, 2000; Licata et al., 2020; Szklarczyk et al., 2019). KEGG was used for identifying LPS-mediated inflammatory networks. The SIGNOR database provides pathways with general classifications, such as ‘macrophage polarization’ or ‘toll-like receptor pathway’ (Licata et al., 2020). The STRING database provides sets of PPIs collected from the scientific literature, as well as genes that are annotated as targets for butyrate (Szklarczyk et al., 2019). Cytoscape (Shannon et al., 2003) was used to merge networks from KEGG and SIGNOR. The STRING database provides a list of genes interacting with the target gene in the descending order that is from the most probable interaction based on its methodology. The PPIs from STRING were added as edges to the graph using NetworkX. Each PPI was designated as activating or inhibitory based on KEGG annotations or review of relevant literature. The resulting graph was subjected to a minimum path analysis via NetworkX to identify pathways linking each butyrate-sensitive gene to caspase-1. Each pathway was marked as activating or inhibitory based on the number of inhibitory PPIs within a path (e.g. an odd number of inhibitory PPIs was deemed as an inhibitory path, otherwise an even or zero number was designated as activating).

### LPS/TLR4 complex-mediated pathway collected from KEGG, SIGNOR and STRING and visualized by Cytoscape

FFAR2 and HDAC3 pathways were added via extended search in the STRING database. Blue-colored edges (paths) indicate promoting interaction, whereas red-colored edges indicate inhibitory interaction.

### Statistical analysis

All data are presented as mean±s.e.m. Analyses were performed using GraphPad Prism. For single group comparisons, paired or unpaired two-tailed Student's *t*-test or Mann–Whitney test were used as appropriate, and multiple comparisons were performed using one-way ANOVA between groups, with differences identified by post-hoc tests, as shown in corresponding figure legends. *P*<0.05 was considered significant.

## Supplementary Material

Supplementary information
