# Supplementary material for: Monitoring of inflammation using novel biosensor mouse model reveals tissue- and sex-specific responses to Western diet
Source: Dis Model Mech. 2022 Jun 24;15(6):dmm049313. doi: 10.1242/dmm.049313 (PMC9235879; doi:10.1242/dmm.049313)
Supplement: Supplementary information [file dmm-15-049313-s1.pdf]

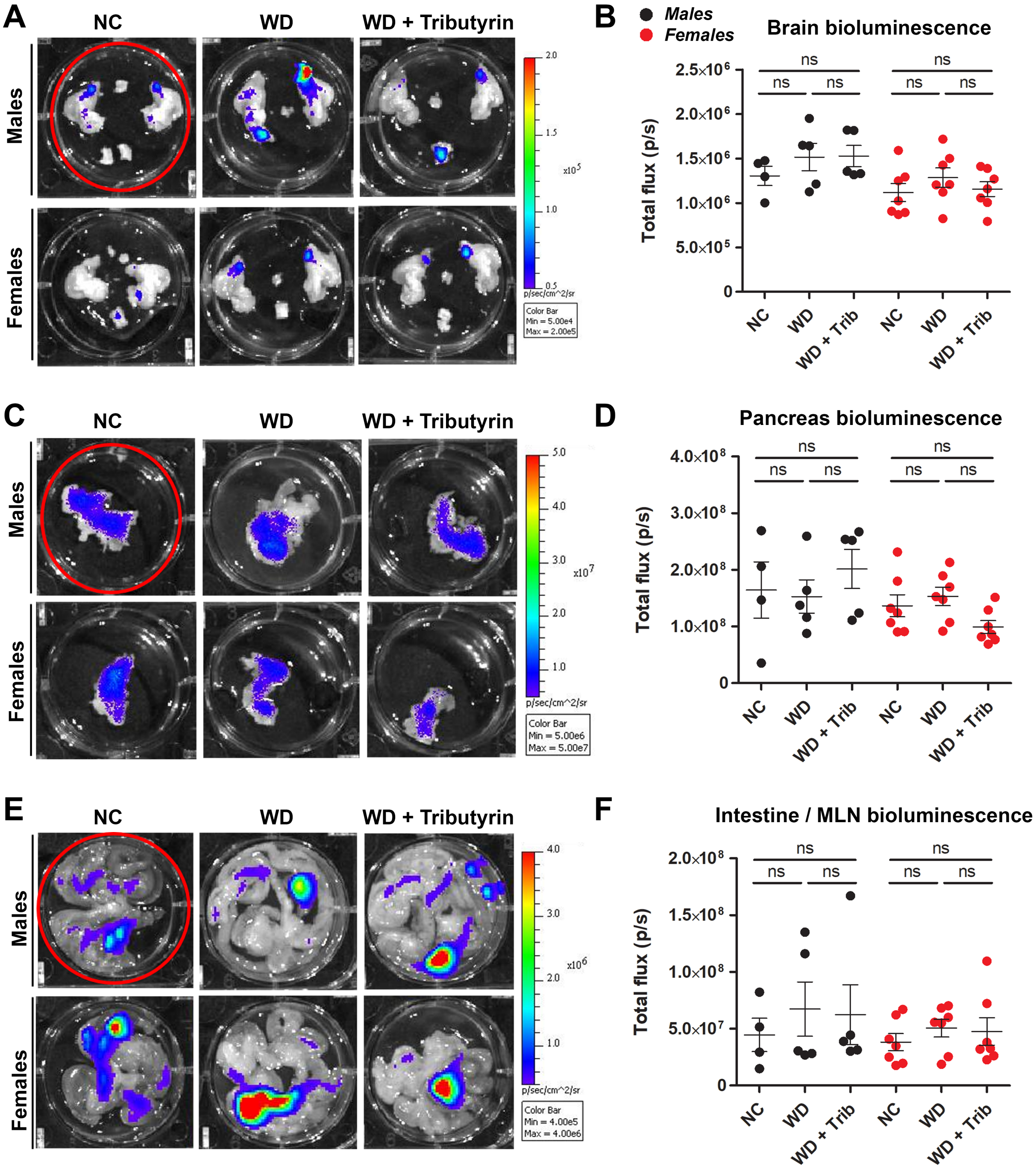
A
NC
WD
WD + Tributyrin
Males
Females
p/sec/cm^2/sr
Color Bar
Min = 5.00e4
Max = 2.00e5
B
Males
Females
Brain bioluminescence
ns
Total flux (p/s)
NC
WD
WD + Trib
C
Color Bar
Min = 5.00e6
Max = 5.00e7
D
Pancreas bioluminescence
E
Color Bar
Min = 4.00e5
Max = 4.00e6
F
Intestine / MLN bioluminescence

Fig. S1. Western diet and tributyrin did not affect caspase-1 activation in the brain, pancreas or intestine.

(A-B) Representative *ex vivo* IVIS images and bioluminescence quantification of brain tissue from normal chow (NC), Western diet (WD), and WD-fed tributyrin treated male and female mice. (C-D) Representative *ex vivo* IVIS images and bioluminescence quantification of pancreas tissue from NC, WD, and WD-fed tributyrin treated male and female mice. (E-F) Representative *ex vivo* IVIS images and bioluminescence quantification of the intestinal tissue, including mesenteric lymph nodes (MLN), from NC, WD, and WD-fed tributyrin treated male and female mice. One-way ANOVA and Tukey's post-hoc. All values represent mean +/-SEM. *p<0.05, **p<0.01, ***p<0.001, n = 4-8 mice/group for males and females.
